# Lunate bone excision and scaphocapitate arthrodesis in late stages of Kienböck’s disease: a long-term prospective study

**DOI:** 10.1007/s00264-025-06458-8

**Published:** 2025-03-01

**Authors:** Amro A. Fouaad, Galal Hegazy, Mohammed Alnahas, Gamal ElSawy, Yasser Saqr, Elsayed Shaheen, Mohamed Gamal, Mohamed Nasr Akl, Ahmed Darweash

**Affiliations:** 1 Al Azhar University, Cairo, Egypt; 2 Portsaid University, Portsaid, Egypt; 3 Suez University, Suez, Egypt

**Keywords:** Kienböck's disease, Scaphocapitate arthrodesis, Lunate excision, Wrist function, Postoperative outcomes

## Abstract

**Purpose:**

This study aims to evaluate the outcomes of scaphocapitate arthrodesis with lunate excision in patients with stage IIIB and IIIC Kienböck’s disease.

**Method:**

Between September 2013 and April 2024, 106 consecutive patients were screened, with 64 consenting to participate. Final analysis included 56 patients (32 stage IIIB and 24 stage IIIC) who underwent scaphocapitate arthrodesis with lunate excision, utilizing distal radius bone grafting stabilized by Herbert compression screws. Preoperative and postoperative assessments (6, 18, 36, and 84 months) included VAS score for pain, ROM, grip strength, MMWS, PRWE scores, and radiographic evaluations including RS angle, CHR, CUDR, and ulnar variance.

**Results:**

The mean operative time was 75 ± 11 min, and the average follow-up was 86 ± 2.5 months. The union rate was 91% with a mean time to union of 10 ± 2 weeks. Preoperative mean VAS scores (63 ± 4 mm) significantly decreased to 25 ± 9 mm at 6 months and 12 ± 4 mm at 36 months (*p* = 0.001), with a slight increase to 22 ± 5 mm at 84 months. ROM improved from 46% ± 9% of the healthy side preoperatively to 59% ± 3.2% at 36 months (*p* = 0.001) but slightly decreased to 58% ± 3% at 84 months. Grip strength improved from 48% ± 8% preoperatively to 89% ± 6.4% at 36 months (*p* = 0.001) and remained stable at 88% ± 4% at 84 months. The mean MMWS increased from 46 ± 7 to 75 ± 5 (*p* = 0.001), while PRWE scores decreased from 68 ± 8 to 23 ± 6 (*p* = 0.001). The mean RS angle decreased from 59° ± 8° preoperatively to 50° ± 3° at 36 months (*p* = 0.001). There was no significant change in CHR (0.44 ± 0.04 to 0.46 ± 0.03, *p* = 0.251), while CUDR decreased from 31 ± 3 mm to 25 ± 2 mm (*p* = 0.021). Ulnar variance remained stable (*p* = 0.325). Degenerative changes were noted in 13 patients (23%) at the RS joint, with six showing Grade I, 5 Grade II, and 1 Grade III degeneration. Additionally, 5 patients (9%) exhibited changes at the STT joint, comprising three with Grade I and 2 with Grade II degeneration.

**Conclusion:**

Scaphocapitate arthrodesis with lunate excision can improves pain, ROM, grip strength, and functional scores in patients with stage IIIB and IIIC Kienböck’s disease. Over time, improvements in VAS scores and functional metrics were notable, though there was a slight decline in pain relief and ROM at 84 months. These changes are critical to understanding the potential degenerative complications, particularly at the RS joint, where some patients developed osteoarthritis.

**Level of evidence:**

Level II.

## Introduction

Kienböck’s disease is a progressive disorder marked by avascular necrosis of the lunate bone. The condition results in wrist pain, reduced range of motion (ROM), and functional impairment [1–4]. As the disease advances through the Lichtman classification system (Table 1) [5], particularly in stages IIIB and IIIC, the structural integrity of the lunate collapses, causing carpal instability and secondary degenerative changes in the surrounding joints. These changes necessitate surgical intervention to alleviate symptoms and maintain wrist function [6].

**Table 1:** The modified Lichtman classification

Stage		Definition
I		Displays normal radiographs, although MRI may reveal increased T2 signals
II		Increased lunate bone density but without alteration in shape or fractures
III	A	Lunate collapse without carpal misalignment (RS angle ranges between 30 and 60 degrees)
	B	Lunate collapse with decreased carpal height (RS angle > 60 degrees)
	C	Coronal fracture of the lunate
IV		Advanced disease with significant carpal arthritis

One of the surgical options for managing advanced Kienböck’s disease is scaphocapitate (SC) arthrodesis. This technique aims to stabilize the wrist by fusing the scaphoid and capitate bones, effectively redistributing load transmission and preserving some degree of wrist motion [7]. While studies have shown favorable outcomes with SC arthrodesis, there remains an ongoing debate about the role of lunate excision as part of this procedure. Advocates for lunate excision argue that removing the necrotic lunate eliminates a source of persistent pain, reduces the risk of progressive collapse [2].

Several studies have reported improved pain relief and function following SC arthrodesis with concurrent lunate excision, particularly in patients with advanced disease stages. However, concerns about this approach include the potential for carpal height reduction, altered wrist biomechanics, and long-term degenerative changes, particularly at the radio-scaphoid (RS), and scaphotrapeziotrapezoid (STT) joint [8]. Conversely, proponents of preserving the lunate during SC arthrodesis suggest that retention of the lunate can maintain more natural carpal kinematics, potentially reducing the risk of adjacent joint arthritis [6, 7]. Some studies have demonstrated satisfactory functional outcomes with this approach, particularly in terms of range of motion (ROM) and grip strength, suggesting that lunate preservation may offer benefits in specific patient populations [8].

Despite these findings, the controversy persists due to the lack of long-term comparative data and the varied methodologies across studies, which makes it challenging to draw definitive conclusions. Given the conflicting evidence, there is a need for more comprehensive, long-term prospective studies to evaluate the outcomes of SC arthrodesis with lunate excision. This study aims to address this gap by providing robust long-term data, focusing on pain relief, functional outcomes, radiographic changes, and complication rates over an extended follow-up period. We hypothesize that SC arthrodesis with lunate excision will result in significant pain reduction, improved grip strength, and functional outcomes in patients with advanced Kienböck’s disease over a long-term follow-up period.

## Materials and methods

**Fig. 1:**
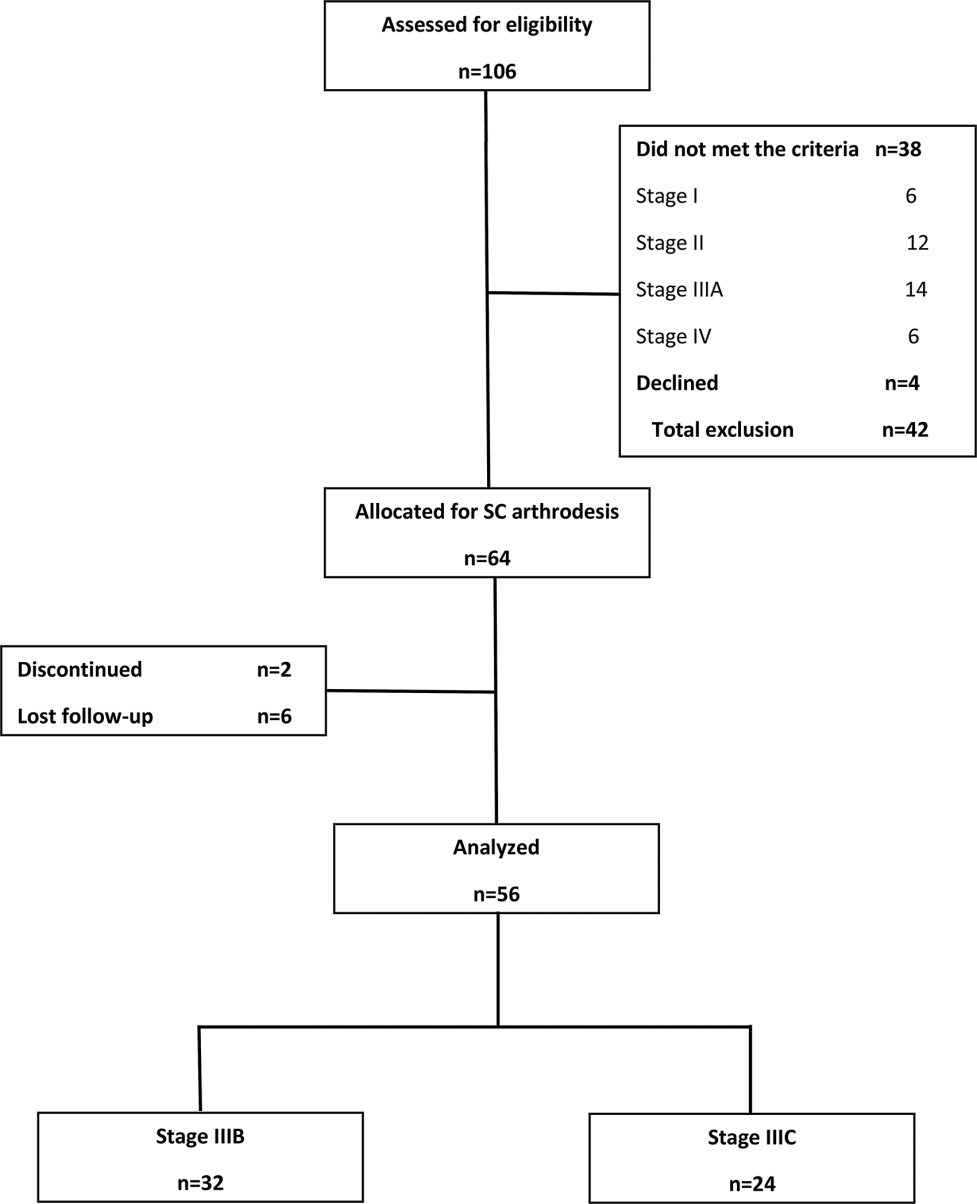
The study flowchart displays the count of included, excluded, and ultimately analyzed patients

The study was approved by our institutional review board before commencement, in accordance with the guidelines of the Helsinki Declaration of 1975, as revised in 2000 and 2008. From September 2013 to April 2024, 106 consecutive patients diagnosed with Kienböck’s disease were enrolled at our university hospitals’ hand surgery clinic. Patients underwent evaluation to determine their eligibility for the study. Inclusion criteria included individuals over 18 years old with stage IIIB or IIIC Kienböck’s disease. Exclusions encompassed patients with less than 36 months of follow-up. Diagnoses were confirmed using standard posteroanterior and lateral wrist radiographs, along with MRI and CT scans. Forty-two patients did not meet the criteria or declined to participate. The remaining 64 patients provided informed consent and underwent treatment discussions. Following surgery, two patients discontinued treatment and six were lost to follow-up. The final analysis comprised 56 patients with stage IIIB (*n* = 32) and IIIC (*n* = 24) Kienböck’s disease as depicted in the study flowchart (Fig. 1). Patients demographics are listed in Table 2.

**Table 2:** Patients’ demographics

Item	Results
Age in years (Mean [SD])	35 ± 8
Stage of the disease (IIIB/IIIC), n	32/24
Gender (Male/Female), n	40/16
Occupation (Manual worker/office workers/student/wife/ doctor/ nurse/ engineer/soldier), n	29/11/3/7/1//2/2/1
Affected side (Right/Left), n	37/19
Dominant/nondominant, n	35/21
Smoking (yes/no), n	15/41

Data are reported as mean ± SD with a 95% confidence interval

Before surgery and at 6, 18, 36, and 84 months postoperatively, pain levels were assessed using the Visual Analogue Scale (VAS) score. This scale consists of a horizontal line ranging from 0 to 100 mm. Based on the VAS score, pain is categorised as follows: a score of 0 indicates no pain; ≤ 19 mm very mild, VAS scores between 20 mm and 35 mm correspond to mild interference with functioning; scores between 35 mm and 60 mm indicate moderate interference; and scores of ≥ 60 mm signify severe interference [9].

**Fig. 2:**
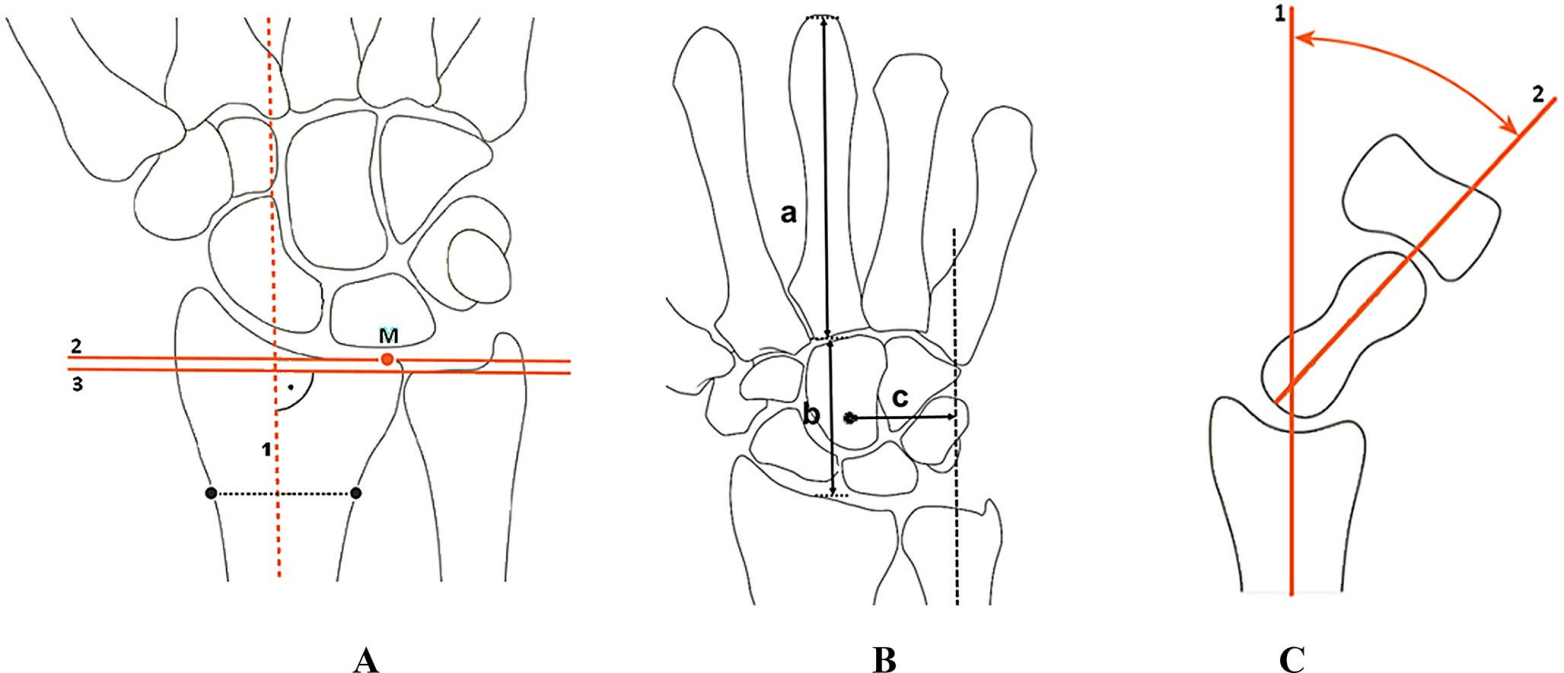
Diagram illustrating the measurement of: **a** The ulnar variance is assessed by drawing a line perpendicular to the long axis of the radius at its distal end and another perpendicular line from the most distal point of the ulnar head to this reference line. The distance between the two perpendicular lines is measured to determine ulnar variance.**b** The diagram depicts the CUDR as the distance from the ulnar axis to the center of the capitate. The CHR is represented as the ratio of the carpal height: **c** The RS angle is defined as the angle formed between two lines: one drawn from the radial styloid to the center of the scaphoid and another from the center of the scaphoid to the center of the capitate

The total ROM, encompassing flexion-extension, radial-ulnar deviation, and pronation-supination, was assessed using a two-hand goniometer and expressed as a percentage of the healthy side [10]. The single maximal effort of grip strength was evaluated using a Jamar hand dynamometer, with results expressed as a percentage of the healthy side, adjusted for hand dominance [11]. Additionally, the Modified Mayo Wrist Score (MMWS) [12], and the patient rated wrist evaluation (PRWE) scores [13] were recorded. The radiographic assessment [14] focused on the ulnar variance as measured by perpendiculars (Fig. 2a), carpal ulnar distance ratio (CUDR) and carpal height ratio (CHR) according to Youm method (Fig. 2b), and RS angle (Fig. 2c). Before surgery, three independent orthopaedic surgeons, each possessing level 3 experience [15], performed both clinical and radiographic evaluations. Following the procedure, another three independent orthopaedic surgeons with level 3 experience conducted the postoperative evaluations.

### Surgical technique

The surgeries were performed under general or regional anaesthesia with an upper arm pneumatic tourniquet while the patient was lying supine. The procedure was conducted by a senior author with level 4 experience or under his supervision. A mid dorsal skin incision starting from the base of third metacarpal to 1 cm proximal to the dorsal ridge of distal radius. The extensor retinaculum was incised between the third and fourth extensor compartments and the posterior interosseous nerve was identified and denervated. Inspection of the carpal bones and its articular surfaces was done via a longitudinal incision of the dorsal joint capsule. A lunate excision was then performed, followed by the preparation of the articular surfaces between the scaphoid and the capitate. The articular cartilage was removed except for the volar rim to maintain the space between the scaphoid and capitate bones. K-wires were used as joysticks to correct carpal misalignment, allowing the scaphoid to be released and derotated. K-wires temporarily stabilised the SC joint to maintain the repositioned scaphoid bone. A cancellous bone graft was taken from the distal end of the radius and inserted into the prepared site. The SC joint was fixid under the guidance of image intensification using two 3-mm Herbert Compression Screws (HCS) (Zimmer^®^), without compressing the SC joint. After deflating the tourniquet and ensuring haemostasis, the wound was irrigated and closed layer by layer. A sterile dressing was applied, and the limb was immobilised in a short-arm thumb plaster splint with the wrist positioned in slight extension and neutral deviation.

## Follow-up

**Fig. 3:**
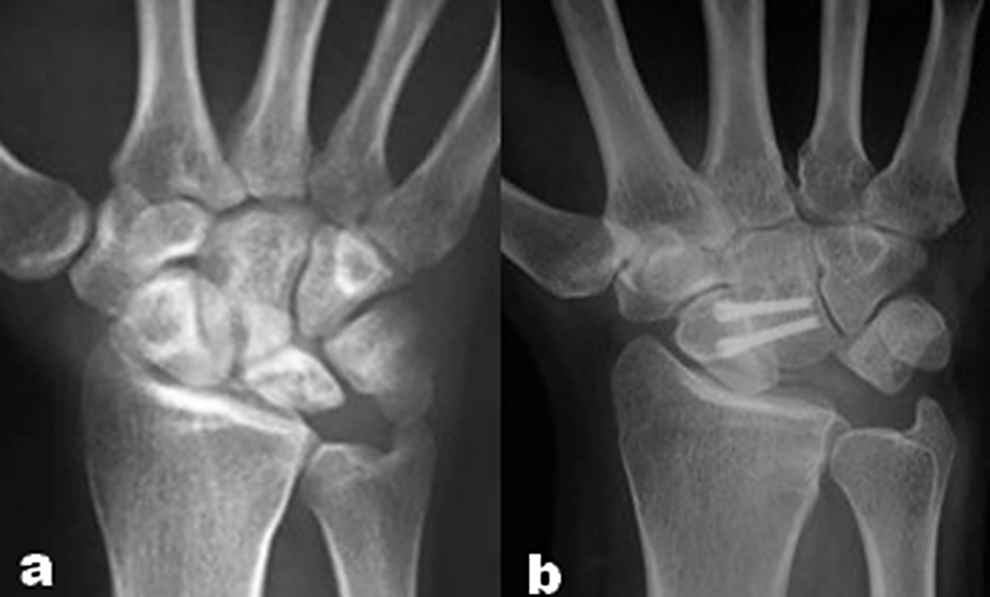
Posteroanterior radiographs of the wrist: (**a**) Preoperative image showing advanced osteonecrosis of the lunate consistent with stage IIIB Kienböck’s disease; (**b**) 18-month postoperative image demonstrating scaphocapitate arthrodesis with excision of the necrotic lunate, improved carpal alignment, and stable fixation

Postoperative management included pain control medication and hand elevation to reduce swelling in the hand and wrist. Sutures were removed at the end of the second week, followed by the application of a waterproof fiberglass thumb spica cast, which was maintained for six weeks. After this period, patients were provided with a removable splint until the arthrodesis site was confirmed to have united. A structured regimen of hand, wrist, elbow, and shoulder exercises commenced immediately after surgery, guided by a specialized hand physiotherapist. Radiographic follow-ups, including standard posteroanterior and lateral wrist views, were conducted at two-week intervals to monitor the SC arthrodesis site for union. Union was defined by the absence of gaps or lucency at the arthrodesis site, with visible trabecular bone bridging (Fig. 3). If union was not clearly established, a CT scan was performed every three weeks. Nonunion was diagnosed when less than 50% of trabecular bone bridging was observed on CT scans at 24 weeks postoperatively. Patients with office jobs were allowed to resume work gradually while still in the cast, whereas manual laborers were advised to return to work only after union was confirmed, with activities limited by pain tolerance. Full return to work and unrestricted activities was permitted once union was achieved and pain was sufficiently managed. The degree of osteoarthritis in the RS and STT joints was assessed using the Kellgren-Lawrence classification [16], which comprises five grades: Grade 0 indicates no osteoarthritis, Grade 1 suggests doubtful osteoarthritis with possible small osteophytes, Grade 2 denotes mild osteoarthritis with definite osteophytes and minimal joint space narrowing, Grade 3 represents moderate osteoarthritis with moderate osteophyte formation and pronounced joint space narrowing, and Grade 4 indicates severe osteoarthritis with large osteophytes and significant joint space narrowing.

### Statistical analysis

The study conducted a sample size estimation to ensure sufficient power for detecting differences in the primary outcome variables: VAS, MMWS, and PRWE scores. To achieve a statistical power of 95% and detect a 10% difference at a significance level of *P* < 0.05, it was calculated that at least 39 patients were needed for the VAS score, 43 for the MMWS score, and 52 for the PRWE score. For the statistical analysis, continuous parametric variables were assessed using the repeated measures ANOVA, while continuous nonparametric variables were evaluated using Friedman’s test. For partially missing data, multiple imputation techniques were used to account for missing values. The results were presented as mean values with standard deviation (SD) and a 95% confidence interval. A p-value of less than 0.05 was considered to indicate statistical significance.

## Results

The mean operative time was 75 ± 11 min, with an average follow-up of 86 ± 2.5 months. The union rate at the arthrodesis site was 91%, with 51 out of 56 patients achieving union and a mean time to union of 10 ± 2 weeks.

**Fig. 4:**
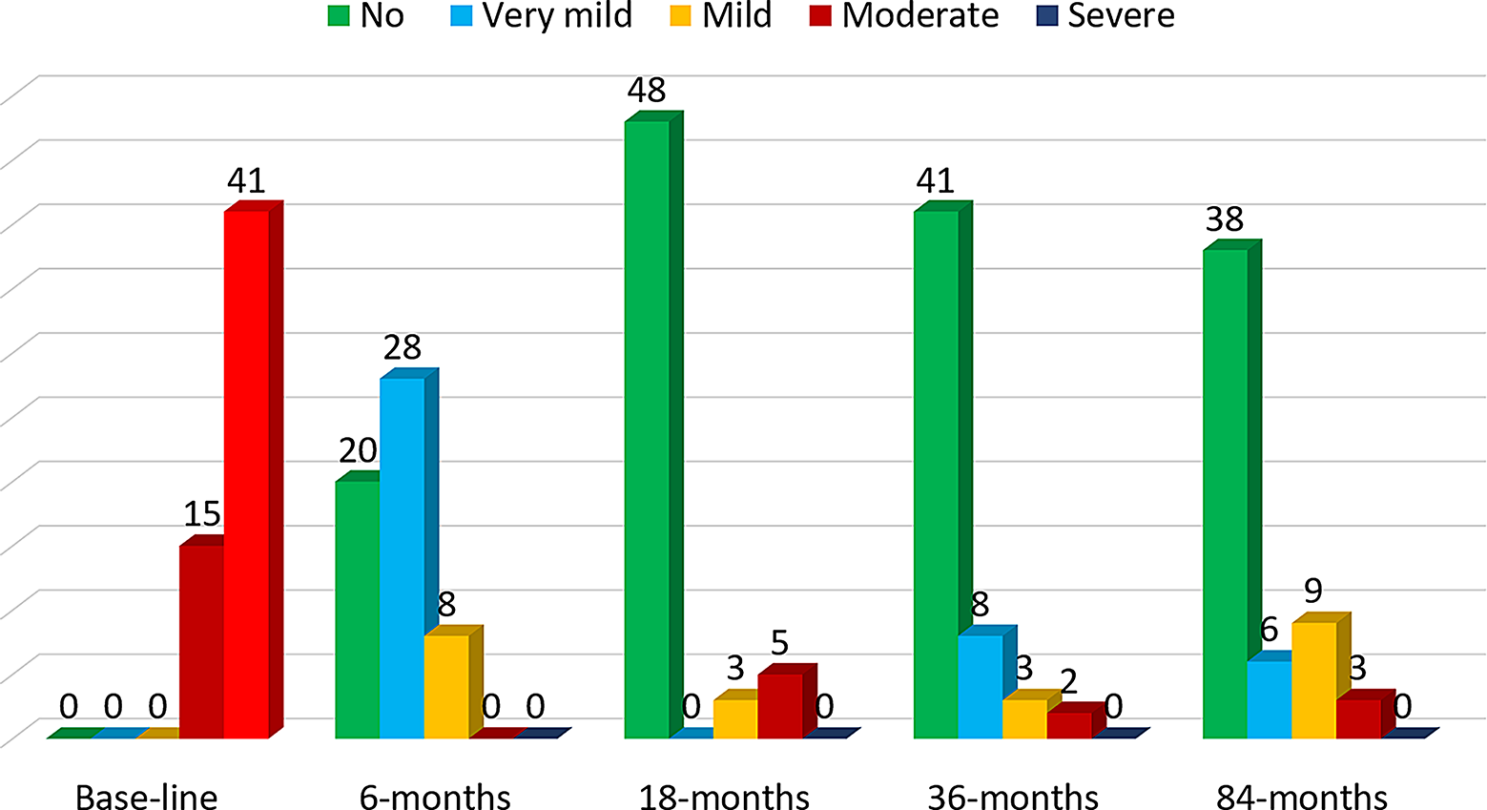
Levels of pain experienced by patients over follow-up intervals from base-line to the last follow-up

Pain Assessment: At the preoperative assessment, patients reported high mean VAS scores, indicating significant pain levels. Postoperatively, there was a significant reduction in pain levels over time compared to preoperative values (*p* = 0.001). At six months postoperatively, the mean VAS score decreased substantially, with most patients reporting no pain, and very mild pain. By 18 months, further improvements were observed, with the majority of patients being pain-free. This trend continued at 36 months, where pain levels reached their lowest point, with most patients reporting no pain. However, by 84 months, there was a slight increase in the mean VAS score, with some patients experiencing mild to moderate pain (Table 3) and [Fig. 4].

**Table 3:** VAS score changes over time and complications

Outcome measure	Preoperative (Mean ± SD)	Postoperative (Mean ± SD)				*p* value
	Base-line data	6-months	18-months	36-months	84-months	
VAS score (mm) Pain-Free (n) Very Mild Pain (n) Mild Pain (n) Moderate Pain (n) Severe pain (n)	63 ± 4 0 0 0 15 41	25 ± 9 20 28 8 0 0	16 ± 5 48 0 3 5 0	12 ± 4 41 8 3 2 0	22 ± 5 38 6 9 3 0	0.001*

VAS Visual Analogue Scale, Data are reported as mean ± SD with a 95% confidence interval, *p < 0.05 indicates statistical significance

ROM and grip strength: At the preoperative assessment, patients exhibited significantly reduced ROM and grip strength compared to the healthy side. Postoperative evaluations revealed gradual improvements over time, with a significant increase in ROM compared to preoperative values (*p* = 0.001). At six months postoperatively, there was a slight improvement in ROM. By 18 months, a more substantial enhancement was observed, with ROM exceeding half of the healthy side’s movement. This upward trend continued at 36 months, where ROM peaked, showing the highest levels of postoperative mobility. By 84 months, ROM remained relatively stable compared to preoperative assessments. In terms of grip strength, patients also showed significant improvements over time (*p* = 0.001). At six months postoperatively, grip strength increased noticeably. By 18 months, grip strength reached substantial levels, approaching those of the healthy side. This improvement persisted at 36 months, where grip strength peaked, marking the highest values recorded during follow-up. At 84 months, grip strength remained stable (Table 4).

**Table 4:** Pre-and postoperative clinical outcomes

Outcome measure	Preoperative (Mean ± SD)	Postoperative (Mean ± SD)				*p* value
	Base-line data	6-months	18-months	36-months	84-months	
ROM (% of healthy side)	46% ± 9%	48% ± 6%	58% ± 7%	59% ± 3.2%	58% ± 3%	0.001*
Grip strength (% of healthy side)	48% ± 8%	60% ± 6%	86% ± 7%	89% ± 6.4%	88% ± 4%	0.001*
MMWS	46 ± 7	55 ± 8	70 ± 7	75 ± 4	75 ± 5	0.001*
PRWE	68 ± 8	51 ± 7	23 ± 4.3	21 ± 3.2	23 ± 6	0.001*

ROM: Range of Motion, MMWS Modified Mayo Wrist Score, PRWE: Patient Rated Wrist Evaluation, Data are reported as mean ± SD with a 95% confidence interval, *p < 0.05 indicates statistical significance

Functional scores: Preoperative assessments revealed that the mean MMWS was notably low. In contrast, the PRWE scores were high (Table 4). Postoperatively, the MMWS showed a modest enhancement at six months, with scores reflecting continued improvement by 18 months. The highest levels were recorded at 36 months, and these scores remained stable at 84 months (*p* = 0.001). Similarly, the PRWE exhibited a significant decrease in scores throughout the follow-up period. Notably, there was a substantial reduction in PRWE scores at six months, with further improvement by 18 months. The lowest levels were maintained through 36 months and remained consistent at 84 months (*p* = 0.001). Of the patients, 44 (79%) were able to resume their usual activities regularly. However, 5 patients (9%) reported a reduction in work hours due to intermittent pain during manual tasks, while 4 patients (7%) switched to lighter jobs, and 3 patients (5%) opted for retirement.

**Fig. 5:**
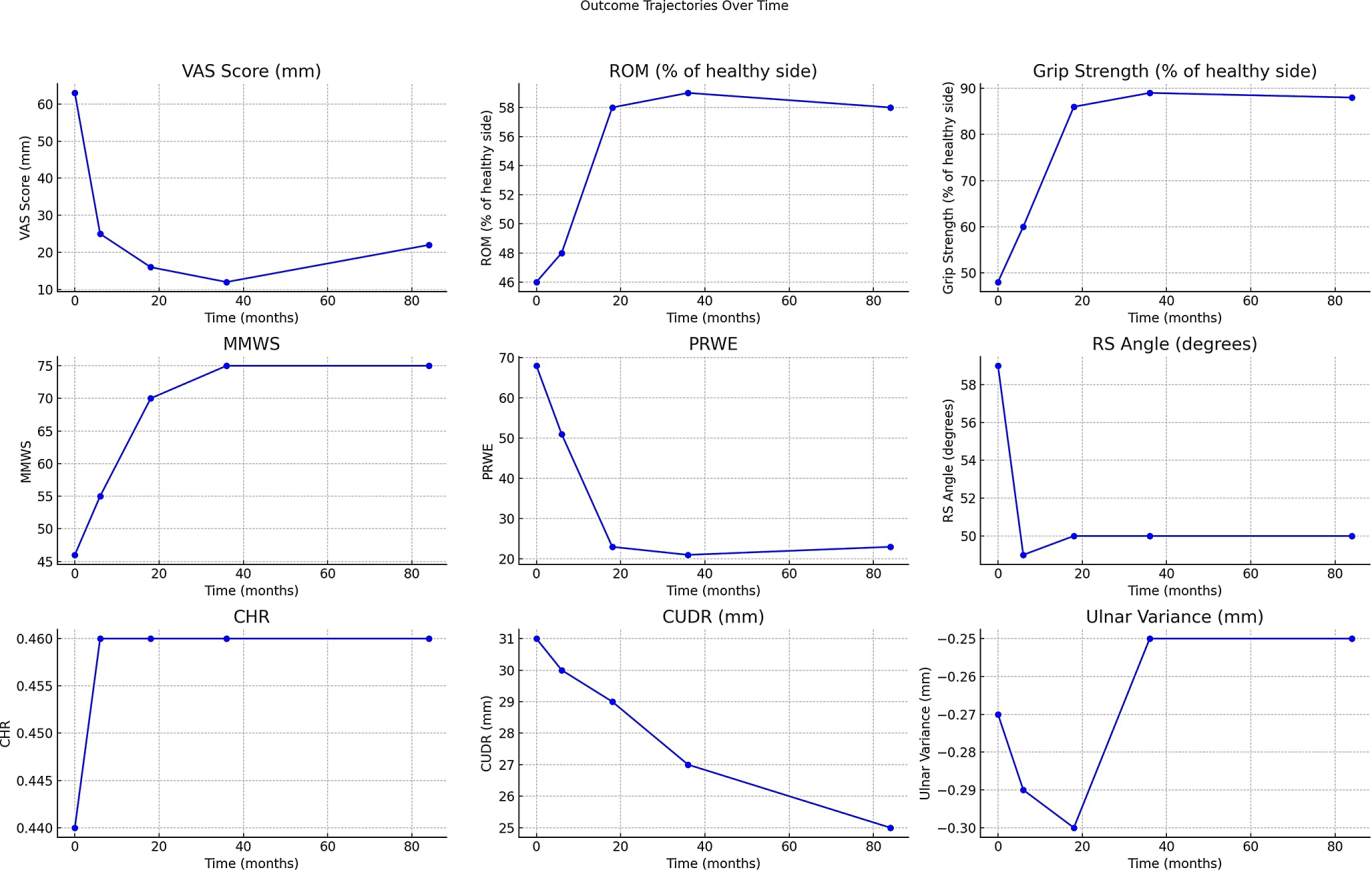
The trajectories of clinical and radiographic outcomes over follow-up intervals. VAS: Visual Analogue Scale, ROM: Range of Motion, MMWS: Modified Mayo Wrist Score, PRWE: Patient Rated Wrist Evaluation, RS: Radioscaphoid angle, CHR: Carpal Height Ratio, CUDR: Carpal Ulnar Distance Ratio

Radiographic outcomes: Preoperatively, the mean RS angle was notably high, while the CHR was lower than optimal. Postoperatively, the mean RS angle demonstrated a marked reduction at six months, which remained consistent through subsequent follow-ups (*p* = 0.001). Conversely, the CHR showed no significant change (*p* = 0.251). The CUDR exhibited a progressive decrease from the preoperative period through to 84 months (*p* = 0.021). The ulnar variance remained stable across all assessments (*p* = 0.325) (Table 5). The progression of clinical and radiographic outcomes and functional scores across follow-up intervals is illustrated in [Fig. 5].

**Table 5:** Pre-and postoperative radiographic outcomes

Outcome measure	Preoperative (Mean ± SD)	Postoperative (Mean ± SD)				*p* value
	Base-line data	6-months	18-months	36-months	84-months	
RS angle (degrees)	59° ± 8°	49° ± 3°	50° ± 4°	50° ± 2°	50° ± 3°	0.001 *
CHR	0.44 ± 0.04	0.46 ± 0.02	0.46 ± 0.03	0.46 ± 0.02	0.46 ± 0.03	0.251
CUDR (mm)	31 ± 3	30 ± 3	29 ± 3	27 ± 4	25 ± 2	0.001 *
ulnar variance (mm)	-0.27 ± 2.96	-0.29 ± 2.90	-0.30 ± 1.95	-0.25 ± 1.96	-0.25 ± 1.90	0.325

RS: Radioscaphoid angle, CHR: Carpal Height Ratio, CUDR: Carpal Ulnar Distance Ratio, Data are reported as mean ± SD with a 95% confidence interval, *p < 0.05 indicates statistical significance

Complications: Five patients who experienced nonunion at the arthrodesis site underwent revision surgeries using iliac bone grafts and staple fixation, which successfully achieved union at an average of 12 weeks (ranging from 11 to 14 weeks) postoperatively. Three patients developed superficial wound infections, which were effectively managed with wound care and antibiotics. Additionally, six patients experienced wrist stiffness, which was treated with pain medication and physiotherapy. Among these, three patients were diagnosed with reflex sympathetic dystrophy, receiving treatment that included nonsteroidal anti-inflammatory drugs, physiotherapy, and bisphosphonates. Arthritic changes were detected in 13 patients (23%) at the RS joint, with six showing Grade I, five Grade II, and one Grade III degeneration. Additionally, five patients (9%) exhibited arthritic changes at the STT joint, comprising three with Grade I and two with Grade II. Among these, five patients experienced impingement of the scaphoid and radial styloid, two patients had radial styloidectomy and three prefer medical treatment for mild to moderate dorsal-radial wrist pain.

## Discussion

SC arthrodesis is recognised as a motion-preserving salvage procedure, primarily intended to prevent further lunate collapse, redistribute carpal loads, and alleviate pain [17, 18]. Pisano et al. [19] initially introduced SC arthrodesis for the management of advanced-stage Kienböck’s disease, with subsequent studies, including that by Sennwald [20], confirming its efficacy.

In our study, SC arthrodesis combined with lunate excision resulted in a significant and sustained reduction in pain. Patients presented with a mean preoperative VAS score of 63 mm, which showed a marked decrease across all follow-ups. By six months postoperatively, the mean score had dropped to 25 mm, with over one-third of patients reporting no pain and none experiencing severe pain, suggesting effective early symptom relief, likely facilitated by wrist denervation and synovial debridement. Pain levels continued to decline, reaching a mean of 16 mm at 18 months and 12 mm at 36 months, with 73% of patients being pain-free. These outcomes are consistent with the findings of Goyal et al. [3], who observed a similar reduction from 73 mm preoperatively to 20 mm at 26 months, reflecting a 72% decrease, comparable to the 80% reduction observed in our cohort. However, at the 84-month follow-up, a slight increase in mean VAS scores to 22 mm was noted, with 10.7% of patients reporting mild pain and 5.4% experiencing moderate pain, possibly indicative of long-term degenerative changes in adjacent carpal joints or altered wrist biomechanics post-surgery. Previous long-term studies, such as Charre et al. [21], have also reported a trend of increasing pain over extended follow-ups, often attributed to adjacent joint degeneration.

Charre et al. [21] evaluated 17 patients who underwent SC arthrodesis with lunate excision, reporting a mean increase in grip strength from 36% preoperatively to 84% postoperatively. In contrast, our study demonstrated a more substantial improvement, with mean grip strength increasing from 48% preoperatively to 88% at the final follow-up. Conversely, Wang et al. [22] performed a retrospective review of 75 patients with stage III Kienböck’s disease, comparing outcomes between patients treated with lunate excision alone and those treated with lunate excision plus tendon ball arthroplasty. They reported postoperative mean grip strengths of 68% and 77% respectively. However, it is essential to note that simple lunate excision can lead to further proximal migration of the capitate, resulting in carpal disorganisation. Interposition materials such as tendon, silicone, or pyrocarbon have yielded inconsistent results in mitigating these complications. These findings suggest that the combination of SC arthrodesis with lunate excision may provide superior grip strength outcomes, as this technique aids in correcting or maintaining scaphoid alignment and carpal height, thereby preserving overall wrist stability.

Studies examining both arthroscopic [1] and open limited intercarpal arthrodesis, whether preserving [2, 3, 23] or excising [21, 24–26] the lunate, consistently reported modest postoperative changes in mean total ROM. Our findings align with this literature, showing a comparable reduction in total ROM to approximately 58% of the unaffected side. Despite the observed decrease in ROM, this cohort study indicates that this limitation does not significantly disrupt the performance of daily activities. This outcome is consistent with expectations, as SC arthrodesis involves the fusion of the scaphoid and capitate bones, inherently limiting movement at the SC joint. While the primary goal of the procedure is to stabilise the wrist and alleviate pain, it naturally leads to reduced flexion, extension, and radial-ulnar deviation, contributing to an overall decrease in ROM.

The significant improvements in grip strength and ROM translated into meaningful clinical outcomes, as reflected in the functional scores. The PRWE score markedly decreased from 68 preoperatively to 23 postoperatively (*p* = 0.001), indicating a reduction in pain and disability. Meanwhile, the MMWS score improved significantly from 46 preoperatively to 75 postoperatively (*p* = 0.001), reflecting enhanced wrist function. Consequently, 44 out of 56 patients (79%) successfully resumed their usual activities within an average recovery period of 31 weeks. These results underscore the procedure’s efficacy in preserving functional outcomes, despite the biomechanical constraints imposed by SC arthrodesis. The findings suggest that the restriction of ROM does not adversely impact the ability to perform daily activities.

The fusion of the scaphoid to the distal carpal row, along with the removal of the lunate, results in increased shear stress at the RS and STT joints. This biomechanical alteration may accelerate the progression of degenerative arthritic changes in these areas. Our findings revealed that 18 patients (32%) exhibited arthritic changes in the RS and/or STT joints, which adversely affected the daily activities of 5 patients (9%), necessitating a second surgical intervention or medical treatment. Nevertheless, despite these adverse effects, the majority of patients with degenerative changes reported satisfaction with their overall outcomes. Similarly, a study by Lee et al. [25] found that 35% of patients undergoing SC arthrodesis presented with degenerative changes at adjacent joints after several years, highlighting that the alteration in wrist biomechanics may predispose these joints to degeneration.

The success rates for union after limited intercarpal arthrodesis range between 80% and 100%, according to existing literature. Charre et al. [21] and Özdemir et al. [27] reported a 100% union rate in their cases, where autogenous bone grafts, taken from the distal radius or iliac crest, were utilized along with various fixation techniques such as multiple K-wires, headless compression screws (HCS), or staples. Similarly, Collon et al. [6] and Lee et al. [25] noted an 80% union rate with the use of K-wire or HCS fixation. Our findings are consistent with these studies, showing a union rate of 91%, with 50 out of 56 patients achieving successful fusion. The use of cancellous bone autografts remains a common practice in the surgical approach to intercarpal arthrodesis, although nonunion cases have been reported across different studies, with varying underlying causes. Our cohort experienced nonunion at the arthrodesis site in 5 pateints (9%). In three patients, the nonunion was attributed to graft resorption or insufficiency, while in two patients, it was due to improper screw placement.

This study demonstrated a consistent improvement in the mean RS angle across follow-ups, decreasing from 59° preoperatively to 50° postoperatively, with no changes observed in the CHR throughout the period. Similar outcomes were reported by Koh et al. [1] and Özdemir et al. [27], who noted reductions in the mean RS angle from 56° before surgery to 51° after SC arthrodesis, without affecting CHR. In contrast, Meena et al. [24] observed a notable decline in CHR, indicating potential progressive carpal collapse postoperatively. The pathophysiology of Kienböck’s disease is believed to be associated with rotatory instability of the scaphoid, leading to increased synovitis, pain, and reduced function. SC arthrodesis, by stabilizing the scaphoid along with lunate excision and synovectomy, proved effective in alleviating pain and enhancing function, irrespective of changes in CHR. Minamikawa et al. [28] supported this, identifying an optimal RS angle range of 30° to 57° that facilitates improved wrist motion following SC arthrodesis based on kinematic studies of cadaveric wrists.

Postoperatively, the CUDR showed a progressive mean reduction from 31 mm preoperatively to 25 mm across follow-ups, suggesting ulnar carpal translation. Despite this decrease, favourable clinical outcomes were maintained, and the carpal–ulnar translation did not significantly impair wrist function. These findings align with those of Park et al. [29], who studied the effects of ulnar carpal translation on function following SC arthrodesis with lunate excision for Kienböck’s disease. They highlighted that weakening of extrinsic wrist ligaments, particularly the radioscaphocapitate (RSC) ligament, could contribute to carpal–ulnar translation. Additionally, Nakamura et al. [30] identified RSC ligament injuries as a possible cause, while Watson et al. [31] suggested prolonged synovitis might predispose to gradual weakening and damage to ligamentous structures. Procedural factors during SC arthrodesis, such as lunate excision, could also increase mechanical stress on the RSC ligament, potentially exacerbating the issue.

This prospective chart demonstrates the SC arthrodesis with lunate excision can reduces pain and improves grip strength, though it is associated with a significant incidence of degenerative changes in adjacent joints over a long follow-up period. Overall, patient satisfaction remains high, underscoring the procedure’s value despite these drawbacks. Future studies should focus on long-term joint health and alternative techniques to mitigate degenerative changes while maintaining functional outcomes.

The study has limitations, including a lack of a control group, which hinders comparisons with alternative treatments, and being a single-center study that may not represent practices across different geographical locations.

### Ethics approval

This study was performed in line with the principles of the Declaration of Helsinki. Approval was granted by the Ethics Committee of our orthopaedic department prior to commencement.

### Consent

Informed consent was obtained from all individual participants included in the study.

## Data Availability

No datasets were generated or analysed during the current study.
